# Lycopene improves testicular damage and sperm quality in experimentally induced varicocele: Relationship with apoptosis, hypoxia, and hyperthermia

**DOI:** 10.1002/fsn3.2762

**Published:** 2022-03-25

**Authors:** Atefeh Babaei, Reza Asadpour, Kamran Mansouri, Adel Sabrivand, Siamak Kazemi‐Darabadi

**Affiliations:** ^1^ 56947 Department of Clinical Sciences Faculty of Veterinary Medicine University of Tabriz Tabriz Iran; ^2^ Medical Biology Research Center Health Technology Institute Kermanshah University of Medical Sciences Kermanshah Iran

**Keywords:** apoptosis, HSPA2, hypoxia, lycopene, varicocele

## Abstract

Varicocele is considered the main reason for male infertility. Antioxidants are common drugs used to reduce the complications of varicocele in these patients. So, we investigated the effects of lycopene on sperm quality, testicular histology, and the expression of some genes in experimentally induced varicocele. Fifty adult male Wistar rats were divided into three groups: control (*n* = 12), sham (*n* = 5), and varicocele (*n* = 33) groups. After 2 months of induced varicocele, five rats were randomly sacrificed and induced varicocele was investigated in each group. Finally, 35 rats were divided into five groups: the control, varicocele, varicocele reserving solvent, and varicocele reserving lycopene (4 and 10 mg/kg) for 2 months. At the end of the experiment, sperm viability, membrane integrity, the expression of Bax, Bcl2, hypoxia (hypoxia‐inducible factor 1α [HIF1‐α]), heat‐shock protein (heat‐shock protein A2 [HSPA2]) genes, and the histology of testes were measured. The results showed a significant decrease in the sperm viability, membrane integrity, Johnson's score, and the expression of the Bcl2 gene in the varicocele group compared to the control group. Also, there was a significant increase in Bax, HSPA2, and HIF1‐α expressions in the varicocele group compared to the control group. Although the administration of lycopene (10 mg/kg) in rats with varicocele improved sperm viability and membrane integrity, Johnson's score, and Bax expression compared to the varicocele group. Our findings indicated that the administration of lycopene in the varicocele group improved sperm quality and testicular injury induced by varicocele via decreasing apoptosis.

## Graphical abstract

Varicocele is considered as the main reason for male infertility and antioxidants are the common drugs in this patient population. However, antioxidant drug therapy for varicocele‐related infertility suffers from a lack of well‐conducted studies providing high evidence. One of the natural powerful antioxidants is lycopene that is the strongest antiradical compound in the carotenoid family. The results of our study showed that the administration of lycopene in the rats with varicocele improved sperm quality and testicular injury.

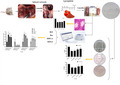

## 1 INTRODUCTION

Varicocele is defined as high pressure, stasis in the spermatic veins, and venous enlargement of the scrotal pampiniform plexus (Yetkin & Ozturk, 2018). The prevalence of varicocele disease is about 35% and 80% with primary and secondary infertility, respectively (Alsaikhan et al., 2016). The relationship between varicocele and infertility is unclear, but several articles have demonstrated that varicocele has caused negative effects on the concentration, motility, viability of sperm, spermatogenesis, Sertoli cellular function, testicular volume, and fertilization (Lara‐Cerrillo et al., 2020; Liang et al., 2015). The hypotheses of varicocele contributing to infertility include hypoxia, heat stress, hormonal imbalances, exogenous toxicants, apoptosis, and oxidative stress (OS) (Agarwal et al., 2012). High levels of reactive oxygen species (ROS) in the semen of men with varicocele have a direct correlation with a decrease in spermatozoa count, motility, morphology, DNA integrity, and germ cell apoptosis (Agarwal et al., 2014).

Another main pathophysiological mechanism of varicocele is testicular hypoxia (Wang et al., 2010). Hypoxia is a deficiency of oxygen in tissue or cells, which occurs due to an abnormal testicular blood flow in varicocele disease (Kumar & Choi, 2015). Mammalian cells activate the hypoxia‐inducible factors (HIFs) in response to hypoxia. HIFs play a crucial role in regulating the transcription of over 100 genes involved in homeostases such as cell survival, cell proliferation, and apoptosis (Wenger et al., 2005). A growing body of research has shown the overexpression of HIF1‐α in patients with varicocele and varicocele animal models (Zhao et al., 2019). The severity of hypoxia determines whether cells become apoptotic or adapt to hypoxia and survive (Greijer & van der Wall, 2004).

Testicular hyperthermia is another pathophysiological mechanism of varicocele (Agarwal et al., 2012). The cells synthesize the heat‐shock proteins (HSPs) which are a group of highly conserved cellular proteins for adaptation to environmental stress such as hyperthermia by the balance between protein synthesis and degradation (Morimoto, 1998). Heat‐shock protein A2 (HSPA2) is a molecular chaperone structurally expressed in the testis (Feng et al., 2001). Several studies have reported that the expression of HSPA2 increased at the transcriptional level in spermatogenic cells and induced apoptosis in testicular spermatogenic cells in the varicocele group compared to the control group (Yu et al., 2021).

Medical management, including the administration of antioxidants, can be a potential low‐risk solution to reduce induced infertility by varicocele (Garg & Kumar, 2016). However, antioxidant drug therapy for varicocele‐related infertility suffers from a lack of well‐conducted studies providing high evidence (Garg & Kumar, 2016). This problem stems from an unspecified treatment goal, poorly designed studies, inadequate measures, and various drug combinations (Garg & Kumar, 2016).

One of the natural powerful antioxidants is lycopene (Rao et al., 2006). Thirteen linear double bonds in the lycopene molecule make it the strongest antiradical compound in the carotenoid family and visualize its biological function (Hedayati et al., 2019). It has been shown that lycopene is twice as effective as β‐carotene and 10 times as effective as α‐tocopherol (Durairajanayagam et al., 2014). It is lipophilic and its sources include tomatoes, papayas, watermelons, apricots, pink grapefruits, and rose hips (Durairajanayagam et al., 2014). The concentration of the lycopene in the testes is 10 times higher than in other tissues (Erdman Jr, 2005). The uneven distribution of lycopene indicates its biological role in the testes (Durairajanayagam et al., 2014). A study on the effects of lycopene on testicular torsion had shown that the administration of lycopene (4 mg/kg) increased sperm motility and protected testis from induced testicular torsion in rats (Hekimoglu et al., 2009). Williams et al. (2020) have demonstrated that the administration of lycopene (14 mg/d) in healthy men improved semen quality such as motility and morphology (Williams et al., 2020). Several studies have indicated that used lycopene in animal semen extender protected sperm from oxidative stress following in vitro storage (Tvrdá et al., 2016; Tvrd et al., 2017). Therefore, we have to conduct more studies with a validated test design with different doses of lycopene to identify its effect on varicocele disease. The present study aimed to investigate the effects of lycopene on sperm quality, testicular histology, the expression of apoptosis, hypoxia, and heat‐shock protein (Hsp) genes in experimentally induced varicocele in rats.

## 2 MATERIAL AND METHOD

### 2.1 Animals

Fifty adult male Wistar rats (180–200 g) were obtained from the animal house of Kermanshah University of Medical Sciences (Kermanshah, Iran). They were kept under standard conditions of controlled light (12:12 h light/dark) and temperature (22 ± 2°C) with free access to standard food and water. The standards for care and use of animals as stated in the ARRIVE (Animal Research: Reporting In Vivo Experiments) guidelines were followed in the present study and all experiments were approved by the Ethical Committee of the University of Tabriz (IR.TABRIZU.REC.1399.041).

### 2.2 Induction of varicocele

**FIGURE 1:**
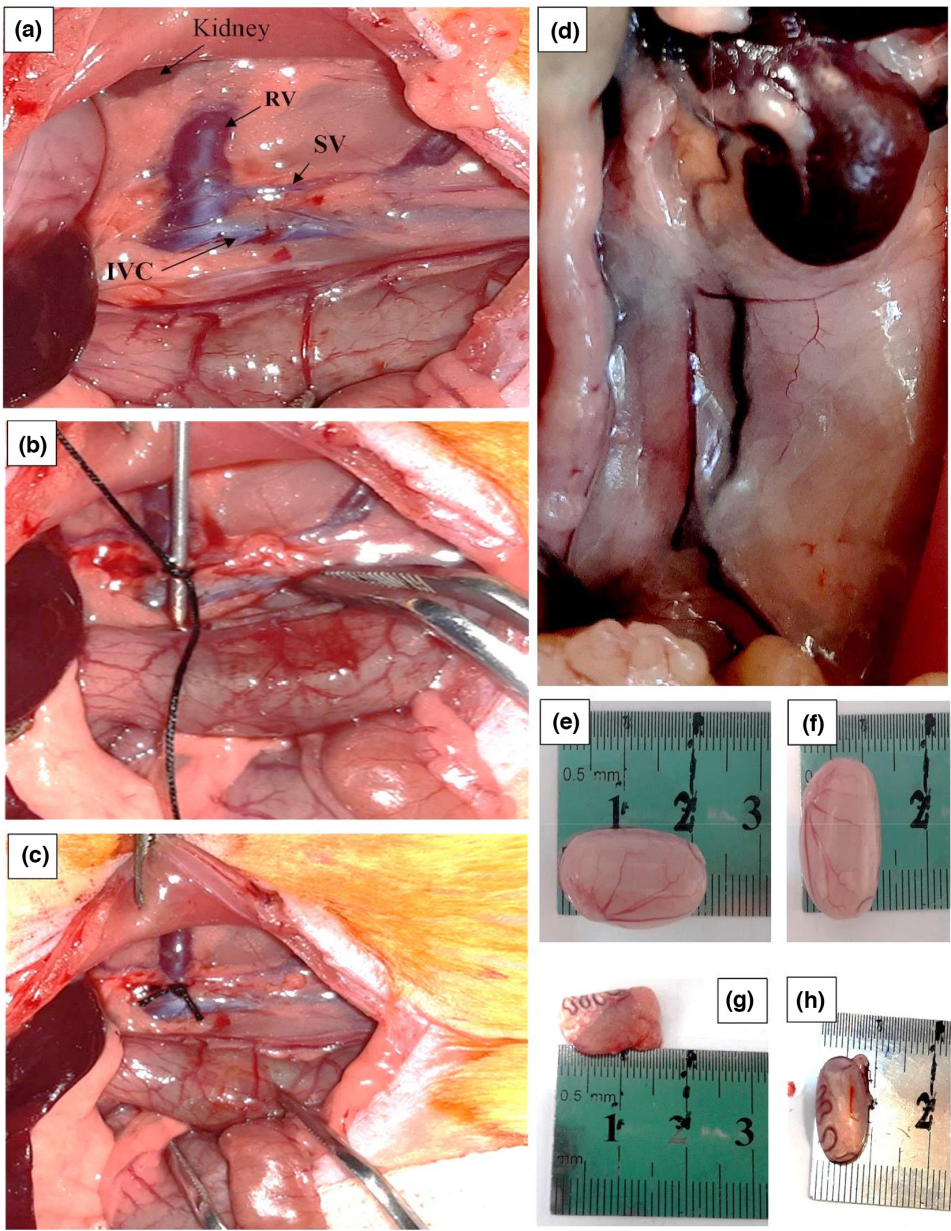
A flowchart of the study design

The 50 rats were randomly divided into control (*n* = 12), sham (*n* = 5), and varicocele (*n* = 33) groups. Unilateral varicocele in the left testis was induced in the latter group following intraperitoneal anesthesia with 75 mg/kg of 10% ketamine (Bremer Pharma) and 5 mg/kg of 2% xylazine (2320 Hoogstraten, Belgium). After shaving and aseptic preparation of the ventral abdominal surface, skin and linea alba incisions (about 3–4 cm) were made in the midline of the abdomen extending from the xiphoid to the pubis. After relocating the internal organs, the left renal vein (LV) was identified and a metal probe (0.8 mm in diameter) was placed parallel to it. An encircling ligature was tied around the vein and metal wire probe using a 4–0 silk suture material nearest to the inferior vein as possible (Figure 1a,b), so that approximately 50% narrowing was observed in the renal vessel. Then, the metal wire probe was removed gently (Figure 1c). Finally, the abdomen was closed in two layers using 3–0 silk suture materials (Katz et al., 2014). The sham group underwent a similar procedure of varicocele induction, except that the left renal vein was dissected free but not ligated. The sham group was designed to investigate the stress caused by the surgical process on the quality of sperm.

### 2.3 Confirmation of induced varicocele

To verify induced varicocele, out of each group, five rats were euthanized randomly with ketamine and xylazine overdose after 2 months. The concentration, motility, viability of sperms, and the weight, length, and width of testis were measured. Previous studies, as well as this study, showed that stress caused by surgery had no effect on rats' sperm quality. As a result, to reduce rat sacrifice, the sham group was removed in the continuation of the study.

### 2.4 Experimental design

After confirming induced varicocele, 35 rats were divided into five subgroups (each group, *n* = 7). (I) Control; (II) Varicocele subgroup, rats with varicocele receiving distilled water via gastric gavage (2 ml, daily for 2 months); (III) Solvent subgroup, rats with varicocele receiving solvent via gavage (2 ml with corn oil, daily for 2 months); (IV and V) Lycopene subgroups, rats with varicocele receiving lycopene (Tinab Shimi, 92%–94%, T50206508, Mashad, Iran) suspension in corn oil via gavage (4 and 10 mg/kg, daily for 2 months).

**FIGURE 2:**
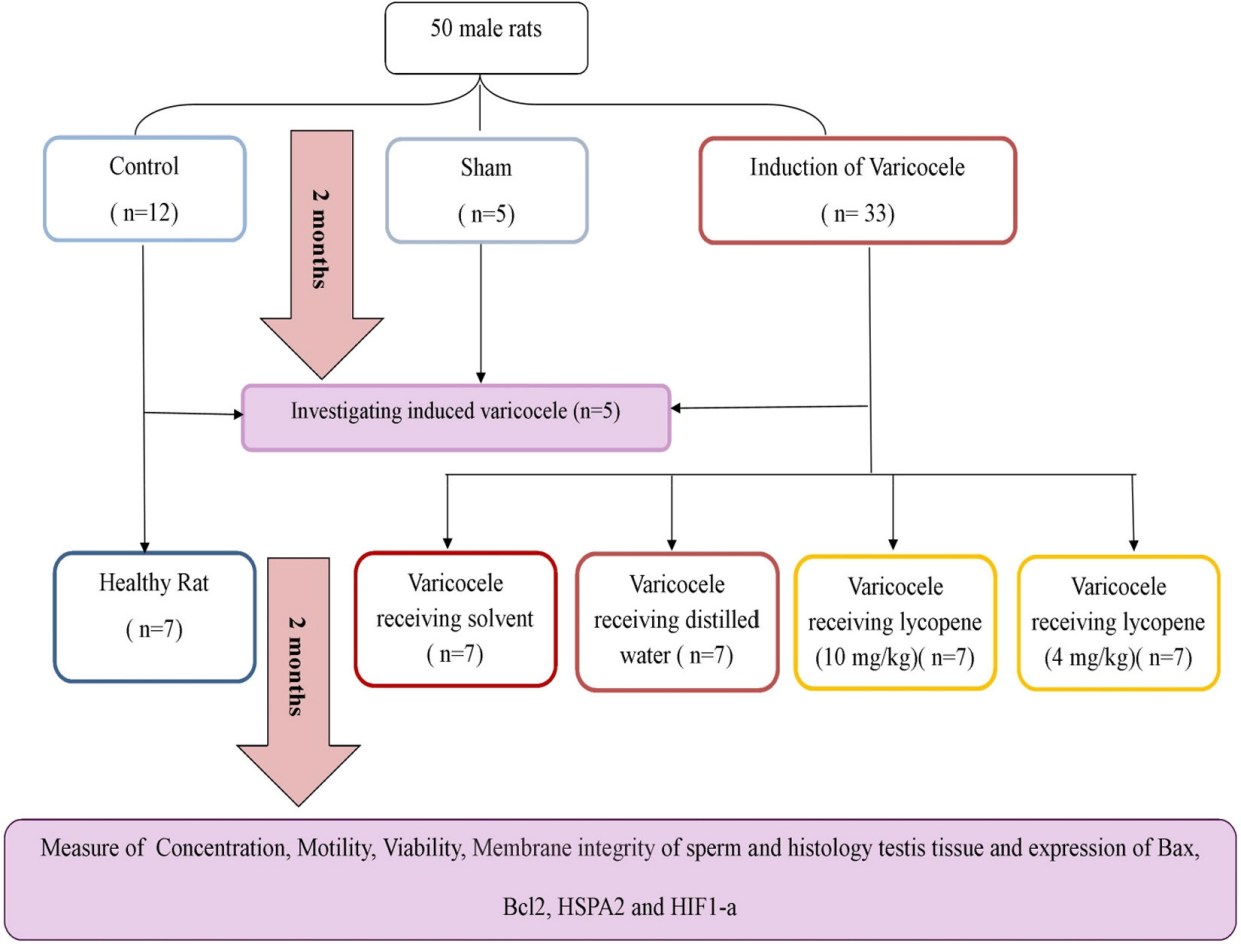
Induced varicocele in the rat. (a) Normal vein; Left renal vein (RV), left spermatic vein (SV), inferior vena cava (IVC); (b and c) partial ligation of the left renal vein; (d) Distended testicular vein in varicocele rats; (e and f) Testis length and width in the control group; (g and h) Testis length and width in the varicocele group after 4 months

The flowchart of the designed experimental is shown in Figure 2.

### 2.5 Sperm collection

After 4 months of varicocele induction (2 months after treatment with lycopene), all the rats were euthanized. The left caudal epididymides were carefully detached from the testis and minced in 5 ml of the modified human tubule fluid (mHTF) (HTF +HEPES (4‐(2‐hydroxyethyl)‐1‐piperazineethanesulfonic acid), Avayeh Tejarat Atiyeh, Tehran, Iran) containing 4 mg/ml of bovine serum albumin (BSA) in a 35‐mm plastic dish at 37°C. Then, epididymides were cut about 10 times using small scissors and incubated at 37°C under 5% CO_2_ for 5 min (Aoto et al., 2011).

### 2.6 Concentration and motility of sperm

Ten microliters of sperm suspension was diluted with 10 μl of distilled water. Then, 10 μl of each sample was transferred into a hemocytometer. The concentration of spermatozoa was counted under a light microscope (Olympus) at ×200 magnification (million/ml). The percentage of sperm motility was measured by placing 5 µl of the sample on a 37°C slide and was counted to be more than 200 spermatozoa in 10 randomly selected fields under a light microscope (Seed et al., 1996).

### 2.7 Sperm viability

To evaluate the percentage of live sperm, 20 µl of sperm suspension was mixed with 20 µl of 0.5% eosin Y (w/v) (1.1535.0100, Merck) solution. A smear was made by placing 10 µl of the mixture on a clean glass slide and allowed to air‐dry. Pink‐stained were counted as dead sperm and unstained were counted as live sperm (200 sperms at a magnification of 400× under a light microscope) (Björndahl et al., 2003).

### 2.8 Membrane integrity

A hypo‐osmotic swelling (HOS) test was used to measure the integrity of the sperm flagella membrane. One hundred microliters of sperm suspension was diluted with 1 ml of warmed hypo‐osmotic solution (0.735 g of sodium citrate dehydrate and 1.351 g of D‐fructose in 100 ml of distilled water) and kept at 37°C for 45 min. Then, 10 µl of suspensions was transferred to a clean slide and covered with a glass coverslip. The number of spermatozoa with coiled tails (intact membrane) and noncoiled tails (damaged membrane) was counted under a light microscope (400×). At least 200 spermatozoa were recorded from each sample (Jeyendran et al.,^1992^).

### 2.9 Histology of testes

At the end of the experiment (4 months), all rats were sacrificed by ketamine and xylazine overdose. The left testis was removed and washed with normal saline. The weight, width, and length of each testis were measured. One half of testis was fixed in Bouin's fixative for 72 h and then, embedded in paraffin. The other half of the samples were stored for gene expression at −70°C. The 5‐μm sections were prepared using a rotary microtome and then, the slides were stained using the hematoxylin and eosin (H&E) staining technique. Three sections from each tissue were provided for histomorphometric analysis. The quality and rate of the spermatogenesis were graded using Johnson's scoring method (Johnsen, 1970) (Table 1). For each animal, 20 cross‐sections from seminiferous tubules were examined and graded. The mean score counts were obtained and compared between groups.

**TABLE 1:** The testis tissue graded based on Johnson's score

Parameters	Score
Complete spermatogenesis with many spermatozoa	10
Many spermatozoa are present but germinal epithelium is disorganized with marked sloughing or obliteration of lumen	9
Only a few spermatozoa are present in section (<5–10)	8
No spermatozoa but many spermatids are present	7
No spermatozoa but only a few spermatids are present	6
No spermatozoa or spermatids are present but many spermatocytes are present	5
Only a few spermatocytes are present	4
Only spermatogonia are present	3
No germ cells are present but Sertoli cells are present	2
No cells in tubular section	1

### 2.10 RNA extraction and complementary DNA (cDNA) synthesis

Total RNA from each tissue was extracted using TRIzol solution (GeneAll, No. 301–001) method according to the manufacturer's instructions. The concentration and the quality of RNAs were evaluated with the absorbance ratios at 260/280 using a NanoDrop spectrophotometer. The synthesis of cDNA was performed according to the protocol of the kit manufacturer (SMOBIO‐ [RP1300] ExcelRT™ Reverse Transcription Kit). Briefly, the cDNA was prepared in a total volume of 20 μl reaction mixture containing 1 μl 10 mM dNTP mix, 1 μl 100 mM random hexamer, 4 μl total RNA, 4 μl 5x reaction buffer, 1 μl RNAOK^TM^ RNAse inhibitor, 1 μl Excel^TM^ Reverse Transcriptase enzyme, and 8 μl diethyl pyrocarbonate (DEPC)‐treated H_2_O, according to the manufacturer's protocol.

### 2.11 Quantitative real‐time polymerase chain reaction (qPCR)

The primers of Bax, Bcl_2_, HIF1‐α, HSPA2, and the glyceraldehyde 3‐phosphate hydrogenase (GAPDH), as internal control genes for rats, are listed in Table 2. The gene‐specific primers were blasted based on the gene sequences of *Rattus norvegicus* presented on the National Center for Biotechnology Information (NCBI homepage) (http://www.ncbi.nlm.nih.gov). The reaction was conducted in a 20 μl volume mix containing 1 μl cDNA, 1 μl forward primer, 1 μl reverse primer, 10 μl SYBR Green qPCR kit (Real‐Time PCR Master Mix Green, Amplicon), and 7 μl distilled–deionized water. Cycling conditions were as follows: general denaturation (95°C, 15 min), which was continued by 37 cycles at 95°C (30 s); annealing (59°C for Bax, Bcl2, and HIF1‐α for 45 s and 58°C for HSP70‐A2 for 45 s); elongation: 72°C (30 s). Each sample was run in duplicate and mean values were calculated, also PCRs without the addition of the template were used as blanks. The expression level of each gene was normalized to GAPDH expression level as the reference gene, and the relative expressions of genes were calculated using the 2^‐ΔΔCt^ method (Livak & Schmittgen, 2001). To obtain the values of fold change from the control group, the relative expressions of the other groups were divided by the relative expression of the corresponding control group (mean =1).

**TABLE 2:** Oligonucleotide sequences for the primers used in real‐time polymerase chain reaction (PCR)

Target gene	GenBank accession no	Forward	Reverse	Amplicon size (bp)
Bcl2	NM_016993.1	ACTTCTCTCGTCGCTACCGTC	AAGAGTTCCTCCACCACCGT	106
Bax	NM_017059.2	CCGGCGAATTGGAGATGAACT	CCAGCCCATGATGGTTCTGAT	229
HIF1‐α	NM_024359.1	CGGAAACTGAAGACCAACAAC	CAGAGGCAGGTAATGGAGACA	114
HSPA2	NM_021863.3	AAAGGTCGTCT GAGCAAGGA	ATAGGACTCCAGGGCGTTTT	117
GAPDH	NM_017008.4	CAATGTGTCCGTCGTGGATCT	GTCCTCAGTGTAGCCCAAGATG	124

### 2.12 Statistical method

All data were analyzed using the SPSS software version 22.0 and the Shapiro–Wilk test was used to assess the normal distribution. All the data had normal distribution and within‐group differences were compared using one‐way analysis of variance (ANOVA) and a post hoc test (Duncan). Collected data were presented as mean ±standard error of the mean (*SEM*), and *p* < .05 was considered to be significant.

## 3 RESULTS

### 3.1 Induced varicocele

To confirm induced varicocele, 2 months after the varicocele induction, five rats in each group were randomly euthanized and the results are presented in Table 3. The varicose veins of the left spermatic vein (LV) and the reduced testes size were visible in varicocele rats (Figure 1d). The body weight changes were not different between groups in the 0 and 60 days (*p* > .05), but after 2 months of varicocele induction, testis weight and width significantly decreased in the varicocele group compared to the control and sham groups (*p* < .05). Sperm parameters including concentration, motility, and the viability of sperm were lower in the varicocele group compared to the control and sham groups (*p* < .05).

**TABLE 3:** Investigated sperm parameters in rats after 2 months of induced varicocele

Groups	BW. Day 0 (g)	BW. day 60 (g)	Testis weight (g)	Testis length (mm)	Testis width (mm)	Concentration (*10^6^)	Motility%	Viability%
Control	189.20 ± 3.65	240.00 ± 11.95	0.98 ± 0.05^a^	18.60 ± 0.93	10.40 ± 0.6^a^	112.00 ± 6.51^a^	76.80 ± 5.13^a^	87.30 ± 2.00^a^
Varicocele	188.80 ± 2.40	231.00 ± 10.47	0.75 ± 0.07^b^	14.80 ± 1.24	8.80 ± 0.66^b^	84.80 ± 7.05^b^	62.70 ± 4.51^b^	70.50 ± 6.15^b^
VCL‐sham	187.60 ± 3.17	246.80 ± 10.07	0.97 ± 0.05^a^	18.20 ± 0.97	9.80 ± 0.66^a^	115.00 ± 7.25^a^	79.30 ± 2.76^a^	85.80 ± 1.93^a^
Sig.	0.93	0.60	0.03	0.05	0.25	0.02	0.04	0.02

Note

All data are given as mean ± *SEM* (*n* = 5). a and b present the significant differences (*p* < .05) between differently marked data.

Abbreviation: BW, bodyweight.

### 3.2 Sperm viability

**FIGURE 3:**
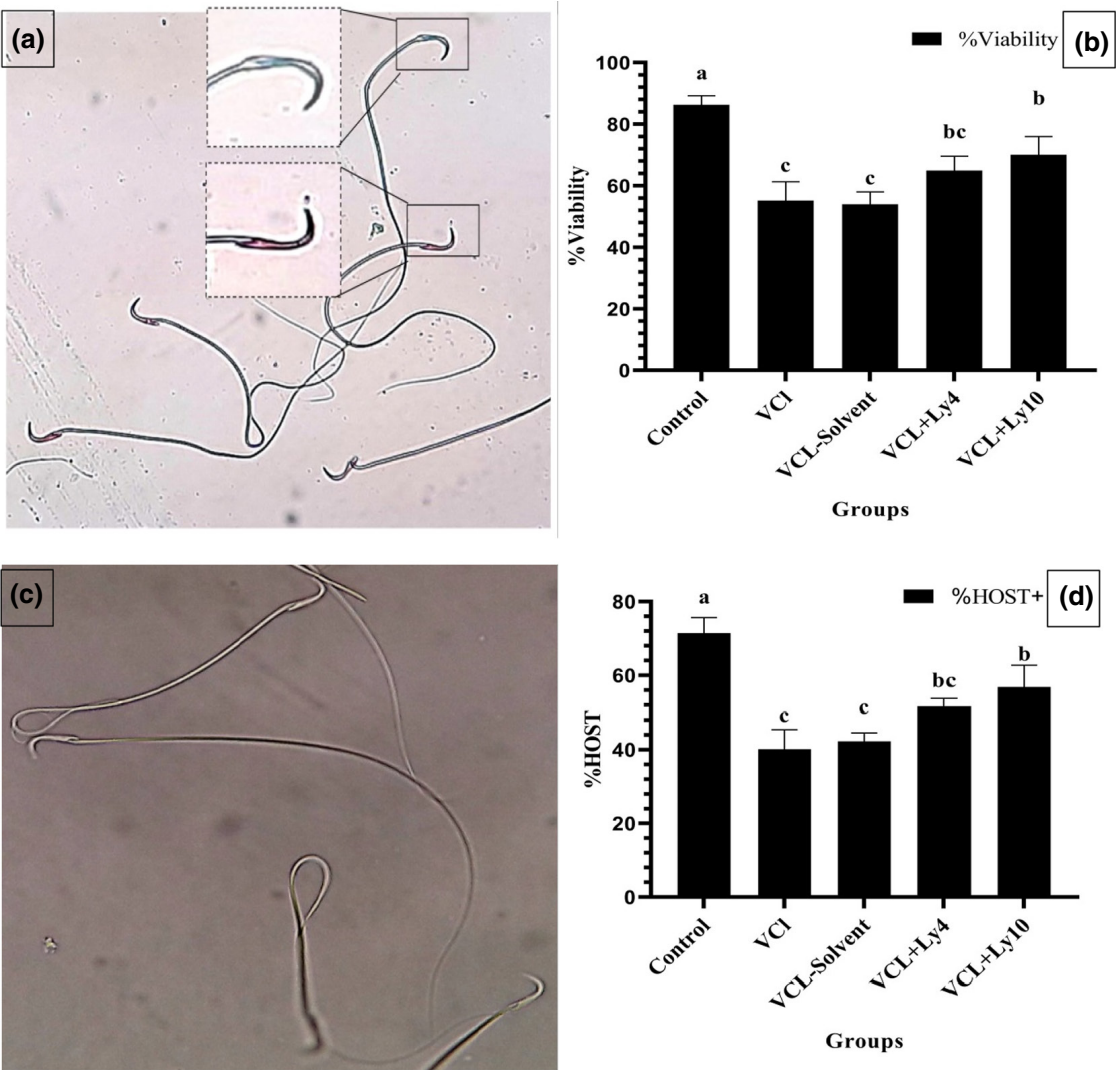
Effect of lycopene on viability (a, b) and membrane integrity (c, d) of sperm in varicocele rats. All data are given as mean ± *MSE* (*n* = 7). (a, b, and c) present significant differences (*p* < .05) between differently marked data

The percentage of sperm viability was measured using eosin staining and spermatozoa with a spectrum of red to pink considered dead (Figure 3a). Based on these results, sperm viability in the control subgroup was higher compared to other subgroups (*p* < .05). On the other hand, the results indicated that the administration of lycopene (10 mg/kg) in rats with varicocele improved viability compared to varicocele and solvent subgroups (*p* < .05).

### 3.3 Membrane integrity

The flagella membrane of intact sperm will be swelling when coming into contact with hypo‐osmotic solution (Figure 3c). The outcome of HOST analysis illustrated that induced varicocele decreased membrane integrity in varicocele rats compared to the healthy ones (*p* < .05) but the administration of lycopene, especially at a dose of 10 mg/kg, protected membrane integrity compared to varicocele and solvent subgroups (*p* < .05) (Figure 3d).

### 3.4 Histological observations

**FIGURE 4:**
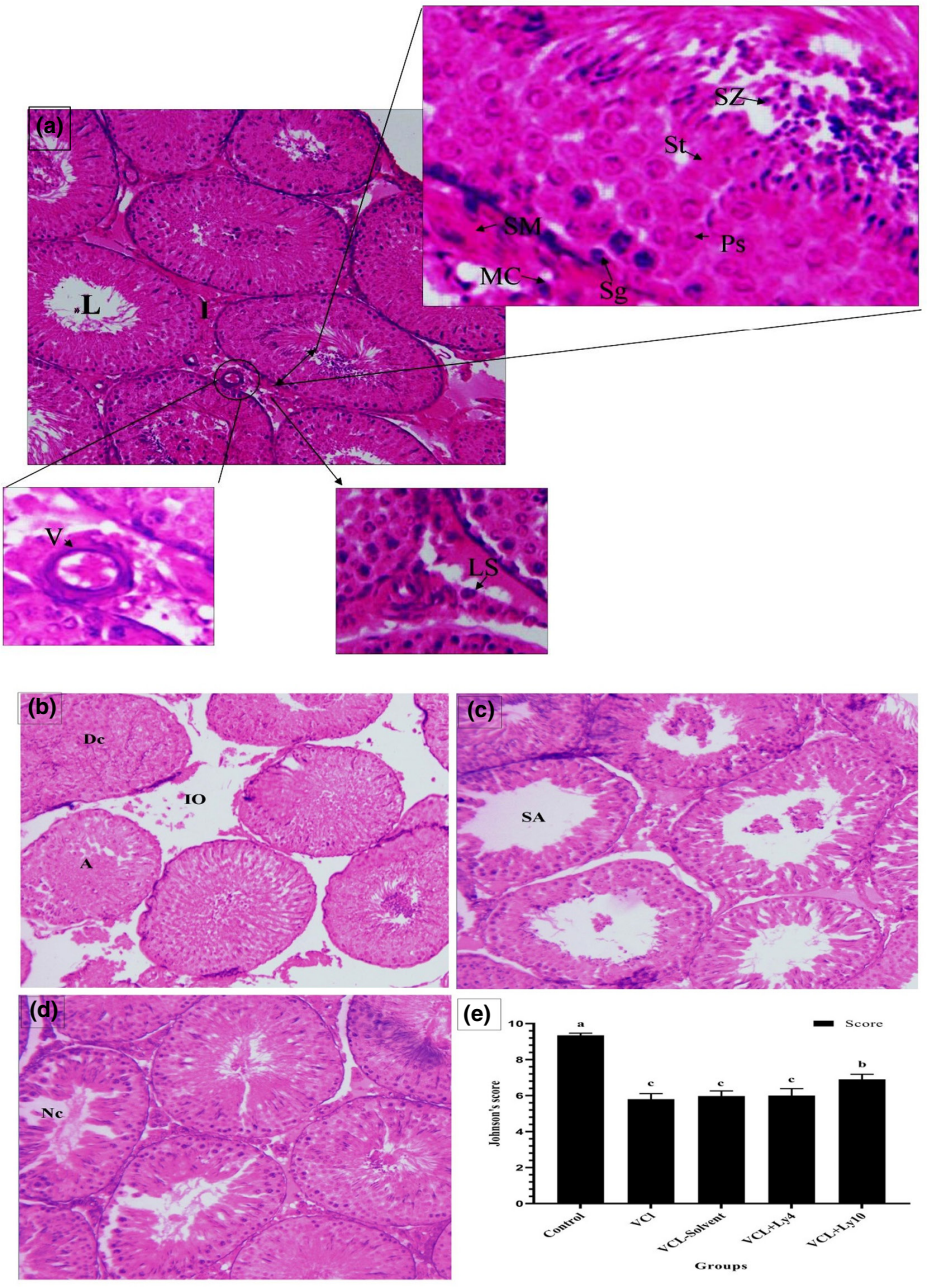
Testis tissues in the four groups were analyzed using the hematoxylin and eosin stain with magnification, ×200 (a: control group, b: varicocele group, c: varicocele receiving lycopene [4 mg/kg], and d: varicocele receiving lycopene [10 mg/kg]) and then were graded based on Johnson's score (e). L, lumen; I, Interstitial tissue; V, vessel; LC, Leydig cells; MC, Myoid cells; SM, Smooth muscle; Sg, spermatogonia; Ps, Primary spermatocytes; St, Spermatid; Sz, Spermatozoa; Dc, Degeneration cells; IO, Interstitial edema; A, Atrophy; SA, Spermatogenesis arrest

Two months after the treatment with lycopene, body weight, and testis length, width, and weight were compared (Table 4). The treatment group and other subgroups were also compared in the mean of Johnson's score (Figure 4e). In the control subgroup, the seminiferous tubule appeared regular and spermatogonia located on the basal lamina, and spermatocytes and spermatids were arranged above them. In the interstitial tissue, blood vessels and Leydig cells were observed (Figure 4a). Comparing subgroups showed that the seminiferous tubule appeared irregular and injured in the varicocele and solvent subgroups (Figure 4b). In untreated subgroups, degeneration and atrophy with severe edema in the interstitial tissue were observed, but their amounts were lower in treated subgroups. In varicocele and solvent subgroups, spermatogenic cells were detected only in a little number of seminiferous tubules, and the interstitial connective tissue was increased compared to other subgroups. The histological studies presented that the administration of lycopene, especially at a dose of 10 mg/kg, reduced the induced injury by varicocele (Figure 4c,d). The results of Johnson's score illustrated that the mean score in the control group was higher than those of other varicocele subgroups (*p* < .05). In the varicocele subgroups, the group reserving lycopene (10 mg/kg) had a significant increase compared to others (*p* < .05) (Figure 4e).

**TABLE 4:** Effect of lycopene on body weight (g) and testes weight (g), length (mm) and width (mm) in the control, varicocele, varicocele reserving lycopene (4 mg/kg) (VCL+Ly4), and (10 mg/kg) (VCL+Ly10) after 4 months

Groups	BW. Day0 (g)	BW. day120 (g)	Testis weight (g)	Testis length (mm)	Testis width (mm)
Control	192.00 ± 2.59	379.14 ± 13.75	1.64 ± 0.04^a^	22.86 ± 0.51^a^	14.00 ± 0.82^a^
Varicocele	191.14 ± 3.25	380.86 ± 11.30	0.78 ± 0.12^c^	16.57 ± 1.19^b^	9.71 ± 1.17^b^
VCL‐solvent	194.43 ± 2.43	395.43 ± 6.69	0.72 ± 0.95^c^	16.57 ± 1.13^b^	9.43 ± 0.48^b^
VCL+Ly4	191.14 ± 5.25	386.57 ± 13.46	1.09 ± 0.07^b^	18.57 ± 0.78^b^	11.14 ± 0.96^ab^
VCL+Ly10	190.86 ± 5.01	382.71 ± 18.60	1.15 ± 0.09^b^	19.43 ± 1.25^b^	12.71 ± 1.19^a^
Sig.	0.965	0.916	0.0001	0.001	0.009

Note

All data are given as mean ± *SEM* (*n* = 7). a, b, and c present significant differences (*p* < .05) between differently marked data.

Abbreviation: BW, bodyweight.

### 3.5 Expression of Bax, Bcl_2_, HIF1‐α, and HSPA2 genes

**FIGURE 5:**
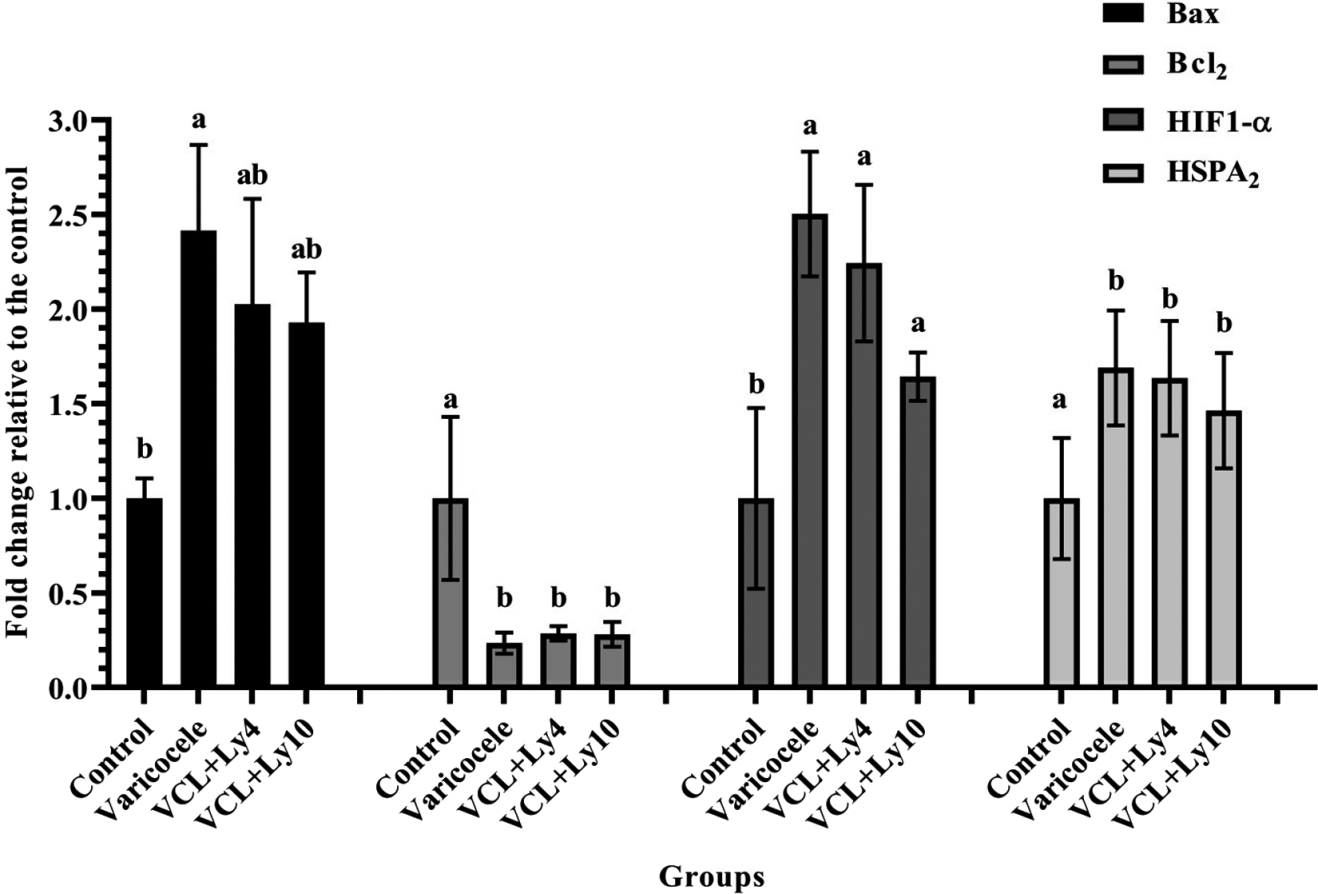
Effect of lycopene on the expression of Bax, Bcl_2_, hypoxia‐inducible factor 1α (HIF1‐α), heat‐shock protein A2 (HSPA2), and glyceraldehyde 3‐phosphate hydrogenase (GAPDH) in control, varicocele, varicocele reserving lycopene (4 mg/kg) (VCL+Ly4), and (10 mg/kg) (VCL+Ly10). All data are given as mean ± *MSE*. a, b, and ab present the significant differences (*p* < .05) between differently marked data

The messenger RNA (mRNA) expression levels of genes were measured by quantitative real‐time PCR. The results of PCR analysis showed that induced varicocele upregulated the expression level of the HIF1‐α in the varicocele and lycopene subgroups compared to the control group (*p* < .05) (Figure 5). On the other hand, the mRNA level of Bcl2 was remarkably higher than in the control subgroup compared to the varicocele and lycopene subgroups (*p* < .05). The investigation of the mRNA level of Bax showed that the groups receiving lycopene (4 and 10 mg/kg) were not significantly different from the control subgroup but the varicocele subgroup was significantly different from the control subgroup (*p* < .05) (Figure 5).

## 4 DISCUSSION

Various studies have identified several mechanisms for the pathophysiology of varicocele disease. The researchers paid more attention to the main mechanisms of induced infertility in varicocele diseases such as hypoxia (Wang et al., 2010), oxidative stress (OS) (Ritchie & Ko, 2021), hyperthermia (Hsiung et al., 1991), and apoptosis (Ammar et al., 2021). Medical management, including the administration of antioxidants, can be a potential low‐risk solution to reduce OS and apoptosis in varicocele‐induced infertility (Garg & Kumar, 2016). Recently, the results of several studies on the effects of natural bio‐actives, such as lycopene, N‐palmitoylethanolamide (PEA), or polydeoxyribonucleotide (PDRN), on experimental varicocele have been promising (Antonuccio, Marini, et al., 2021; Antonuccio et al., 2020; Antonuccio, Micali, et al., 2021). So, the present study attempted to investigate the effects of lycopene, as a powerful antiradical compound in the carotenoid family, on sperm quality, testicular histopathology, and apoptosis, hypoxia, and heat‐shock protein gene expression in experimentally induced varicocele.

This study indicated decreased concentration, motility, and viability of sperm in rats with varicocele after 2 months and also reduction in the same parameters and the membrane integrity of sperm after 4 months compared to healthy rats (*p* < .05). Several studies have reported the same results as those of the present study and documented increased concentration, motility, viability (Hassani‐Bafrani et al., 2019; Najaran et al., 2019), and membrane integrity of sperm in the varicocele disease (Vivas‐Acevedo et al., 2014). The high levels of ROS, however, have a direct correlation with a decrease in spermatozoa count, motility, morphology, and membrane integrity (Agarwal et al., 2014). Due to inadequate cell repair systems, spermatozoa have very little cytoplasmic content and consequently, the insufficient antioxidant content is exposed to OS (Dutta et al., 2019). Spermatozoa contain high levels of polyunsaturated fatty acids (PUFAs) in their plasma membranes and are susceptible to membrane lipid peroxidation (LPO), which reduces membrane fluidity (Agarwal et al., 2017). As a result, the membrane structure of sperm is damaged, leading to reduced motility and fertilization (Cho et al., 2016).

The administration of lycopene in the rats with varicocele, especially at a dose of 10 mg/kg, protected sperms from the reduction of viability, membrane integrity, and testicular damage against the complications of varicocele induction compared to the untreated rats with varicocele. Various studies have investigated the effects of lycopene on fertility in men and animals and showed promising results. They proposed that lycopene could protect sperm from OS by reducing the ROS level and increasing antioxidant enzymatic levels (Babaei, et al., 2021; Babaei, Moradi, et al., 2021; Durairajanayagam et al., 2014; Tripathy et al., 2020). They also explained that this performance of lycopene reduces sperm DNA fragmentation, LPO of the plasma membrane, and finally improves concentration, motility, viability, and morphology in the sperms of humans (Lu‐Lu & Zhi‐Gang, 2020; Williams et al., 2020) and animals (Babaei, Asadpour, et al., 2021; Babaei, Moradi, et al., 2021; Tripathy et al., 2020; Tvrda et al., 2017).

Our finding also indicated that the expressions of HIF1‐α and Bax mRNA were higher and Bcl_2_ was lower in the testis tissues of the rats with varicocele than healthy rats (*p* < .05). Zhao et al. (2019) explained that the expression of HIF1‐α and Bax increased in varicocele rats compared to the control group while Bcl2 was reduced (Zhao et al., 2019). Also, they showed silencing the HIF1‐α gene decreased apoptosis in germ cells and improved the function of spermatogenesis in the varicocele rats (Zhao et al., 2019). This study indicated that the mRNA expression level of the apoptotic gene (Bax) in the groups reserving lycopene (10 and 4 mg/kg) was lower than that of the varicocele group (*p* < .05), which is consistent with the results of the study done about the effects of lycopene in experimental varicocele (Antonuccio et al., 2020). Previous research on the effects of lycopene on fertility documented that lycopene can improve antioxidant capacity and reduce the level of oxidative stress by reducing ROS (Akalin et al., 2016; Tvrdá et al., 2016; Tvrda et al., 2017), so, the level of Bcl2 expression had increased and the level of Bax expression and apoptotic had decreased (Soares et al., 2014; Türk et al., 2010). Perhaps, this is the mechanism that introduces the effect of lycopene in protecting sperm from oxidative stress caused by induced varicocele.

Testicular hyperthermia and hypoxia have an important role in OS‐induced testicular dysfunction in varicocele disease (Makker et al., 2009). The severity of hypoxia determines whether cells become apoptotic or adapt to hypoxia and survive (Babaei, Asadpour, et al., 2021; Babaei, Moradi, et al., 2021; Greijer & van der Wall, 2004). Hypoxia induces apoptosis by inhibiting the electron transport chain at the inner membrane of the mitochondria. The reduction of mitochondrial‐derived adenosine triphosphate (ATP) causes the activation of Bax or Bak, leading to the release of cytochrome C into the cytosol. Cytochrome C binds to the apoptotic protease activating factor 1 (Apaf‐1) (Li et al., 1997). Apaf‐1 activates caspase 9, and then, cleaves caspases 3 and 6, leading to cell death (Li et al., 2004).

One of the processes to protect the cell against the negative effects of physiological stresses such as hyperthermia and hypoxia conditions is the synthesis of a protein family called heat‐shock protein (Hsp) (Flanagan et al., 1995). HSPA2 is structurally expressed in the testes. The expression of HSPA2 in the testis has been shown to occur in two phases: meiosis and spermiogenesis (Feng et al., 2001). Several studies have reported that the expression of HSPA2 increased at the transcriptional level in spermatogenic cells and induced apoptosis in testicular spermatogenic cells in the varicocele group compared to the control group (Khosravanian et al., 2014; Yu et al., 2021). Our finding indicated that the expression of HSP70 was significantly increased in the varicocele group compared to the control group (*p* < .05).

On the other hand, in 2015 Minutoli et al. illustrated that the inhibitors of apoptosis proteins (IAPs) as intrinsic regulators of the caspase cascade are reduced in varicocele rats (Minutoli et al., 2015). Activation of adenosine A2A‐receptors stimulates vascular endothelial growth factor (VEGF) in hypoxia conditions and administration of varicocele rat with polydeoxyribonucleotide can stimulate adenosine A2A‐receptors and may improve depressed testicular function in varicocele (Minutoli et al., 2011). Besides, some studies illustrated that transforming growth factor (TGF)‐β3 is an important cytokine in the regulation of the testicular blood barrier that is involved in testicular damage and infertility in varicocele. Some studies have shown that mitogen‐activated protein kinases (MAPK)‐p38 is a major member of MAPK signaling that is closely related to the degree of oxidative stress and initiates apoptosis. When the Sertoli cell binding barrier is assembled, TGF‐β3 levels are reduced, which play an important role in varicocele‐induced testicular dysfunction (Antonuccio, Marini, et al., 2021; Minutoli et al., 2009). These findings have suggested that in addition to the main known mechanisms, to better understand the pathophysiological mechanism of varicocele, other mediating mechanisms should be examined.

## 5 CONCLUSION

The results of our study showed that the administration of lycopene, as a powerful antioxidant, in the rats with varicocele, especially at a dose of 10 mg/kg, was more efficient in the improvement of sperm functional parameters. Lycopene protected sperm and testicular tissue from induced apoptosis by oxidative stress. So, the concentration, viability, and membrane integrity in lycopene groups were higher than in varicocele groups. However, further assessment is required to show that lycopene induces its effects through the reduction of oxidative stress. On the other hand, testicular environmental alterations caused by varicocele, such as increased temperature, hypoxia, and oxidative stress, may lead to changes in gene expression due to epimutations that have implications in sperm production and fertility. So, the review of the main genetic and epigenetic changes related to varicocele may provide a better understanding of the mechanisms involved in the pathophysiology underlying varicocele development.

## CONFLICT OF INTEREST

The authors declared no potential conflicts of interest to the research, authorship, and/or publication of this article.

## AUTHOR CONTRIBUTIONS

**Atefeh Babaei:** formalAnalysis (equal) ; investigation (equal); methodology (equal); projectAdministration (equal); software (equal); writingOriginalDraft (equal); writingReviewEditing (equal). **Reza Asadpour:** methodology (equal) ; supervision (equal); validation (equal); writingOriginalDraft (equal); writingReviewEditing (equal). **Kamran Mansouri:** projectAdministration (equal) ; supervision (equal); validation (equal); writingReviewEditing (equal). **Adel Sabrivand:** investigation (equal) ; software (equal); writingReviewEditing (equal). **Siamak Kazemi‐Darabadi:** methodology (equal) ; software (equal); writingReviewEditing (equal).

## Data Availability

Datasets generated for this study are available on request to the corresponding author.
